# Programmed death-ligand 1 (PD-L1) characterization of circulating tumor cells (CTCs) in muscle invasive and metastatic bladder cancer patients

**DOI:** 10.1186/s12885-016-2758-3

**Published:** 2016-09-22

**Authors:** Archana Anantharaman, Terence Friedlander, David Lu, Rachel Krupa, Gayatri Premasekharan, Jeffrey Hough, Matthew Edwards, Rosa Paz, Karla Lindquist, Ryon Graf, Adam Jendrisak, Jessica Louw, Lyndsey Dugan, Sarah Baird, Yipeng Wang, Ryan Dittamore, Pamela L. Paris

**Affiliations:** 1 Division of Hematology-Oncology, Helen Diller Family Comprehensive Cancer Center, University of California at San Francisco, 1825 4th Street, 6th Floor, San Francisco, CA 94158 USA; 2 Epic Sciences, San Diego, CA USA; 3 Department of Urology, Helen Diller Family Comprehensive Cancer Center, University of California at San Francisco, San Francisco, CA USA

**Keywords:** Circulating tumor cells, PD-L1, Bladder cancer, Liquid biopsy, Biomarkers

## Abstract

**Background:**

While programmed death 1 (PD-1) and programmed death-ligand 1 (PD-L1) checkpoint inhibitors have activity in a proportion of patients with advanced bladder cancer, strongly predictive and prognostic biomarkers are still lacking. In this study, we evaluated PD-L1 protein expression on circulating tumor cells (CTCs) isolated from patients with muscle invasive (MIBC) and metastatic (mBCa) bladder cancer and explore the prognostic value of CTC PD-L1 expression on clinical outcomes.

**Methods:**

Blood samples from 25 patients with MIBC or mBCa were collected at UCSF and shipped to Epic Sciences. All nucleated cells were subjected to immunofluorescent (IF) staining and CTC identification by fluorescent scanners using algorithmic analysis. Cytokeratin expressing (CK)^+^ and (CK)^−^CTCs (CD45^−^, intact nuclei, morphologically distinct from WBCs) were enumerated. A subset of patient samples underwent genetic characterization by fluorescence in situ hybridization (FISH) and copy number variation (CNV) analysis.

**Results:**

CTCs were detected in 20/25 (80 %) patients, inclusive of CK^+^ CTCs (13/25, 52 %), CK^−^CTCs (14/25, 56 %), CK^+^ CTC Clusters (6/25, 24 %), and apoptotic CTCs (13/25, 52 %). Seven of 25 (28 %) patients had PD-L1^+^ CTCs; 4 of these patients had exclusively CK^−^/CD45^−^/PD-L1^+^ CTCs. A subset of CTCs were secondarily confirmed as bladder cancer via FISH and CNV analysis, which revealed marked genomic instability. Although this study was not powered to evaluate survival, exploratory analyses demonstrated that patients with high PD-L1^+^/CD45^−^CTC burden and low burden of apoptotic CTCs had worse overall survival.

**Conclusions:**

CTCs are detectable in both MIBC and mBCa patients. PD-L1 expression is demonstrated in both CK^+^ and CK^−^CTCs in patients with mBCa, and genomic analysis of these cells supports their tumor origin. Here we demonstrate the ability to identify CTCs in patients with advanced bladder cancer through a minimally invasive process. This may have the potential to guide checkpoint inhibitor immune therapies that have been established to have activity, often with durable responses, in a proportion of these patients.

**Electronic supplementary material:**

The online version of this article (doi:10.1186/s12885-016-2758-3) contains supplementary material, which is available to authorized users.

## Background

Bladder cancer is the 5th most common cancer affecting both men and women in the United States, with a rising incidence worldwide [1, 2] (http://seer.cancer.gov/statfacts/html/urinb.html). The prognosis for patients with muscle invasive (MIBC) and metastatic (mBCa) bladder cancer is poor, with median survival with cisplatin-based chemotherapy averaging 14 months in the metastatic setting [3–6]. Urothelial bladder cancers have been found to express the markers programmed death-1 (PD-1) and programmed death ligand 1 (PD-L1) [7]. Expression of PD-1 and PD-L1 on cancer cells is hypothesized to allow cancers to evade immune surveillance and eradication. The discovery of this mechanism of resistance has provided the rationale for the development of PD-1 and PD-L1 checkpoint immunotherapy.

PD-1 checkpoint immunotherapy is rapidly emerging as a promising option for pre-treated patients with advanced tumors [8–11]. Multiple monoclonal antibodies have been developed against PD-1, and its ligand, PD-L1, and are currently being evaluated in clinical trials either as monotherapy, or in combination with cytotoxic chemotherapy, anti-angiogenic agents, or other immune checkpoint inhibitors [12–14]. Complete responses have been seen in heavily pre-treated patients, with some patients garnering continued tumor regression off therapy [15]. Early phase clinical trials have yielded promising results across many tumor types, measured both by response rate and duration of tumor response [12, 13, 16–18]. As of 2016, immunotherapies targeting PD-1 or PD-L1 have been approved by the FDA for the treatment of relapsed/refractory melanoma [19], squamous cell lung cancer [20, 21], non-small cell lung cancer [22], renal cell carcinoma [23], and most recently bladder cancer [24].

While PD-1/PD-L1 blockade has activity across a number of cancers, in most studies, less than 50 % of patients respond to treatment, indicating a need for predictive biomarkers. While higher PD-1 or PD-L1 expression on tumor biopsy specimens or tumor-infiltrating lymphocytes has been correlated with an increased likelihood of response [7], the positive and negative predictive value of these assays remains modest [13, 14, 25]. In many clinical studies PD-1 and PD-L1 expression has been assessed on archived specimens and may not reflect the current state of the cancer.

Obtaining solid tumor tissue biopsy specimens involves an invasive, technically challenging procedure posing risks to the patient. Instead, circulating tumor cell (CTC) isolation and analysis from peripheral blood samples may provide a fairly non-invasive approach to identify biomarkers and serially monitor response to treatment. Here, we present an assay for PD-L1 protein expression on peripherally collected CTCs [26, 27] and evaluate the incidence of circulating PD-L1^+^ CTCs in blood samples from patients with bladder cancer.

## Methods

### Cell culture and preparation of cell line control slides

Authenticated cell line cells H820 (lung cancer), Colo205 (colon cancer), A549 (lung cancer), SU-DHL-1 (lymphoma), H441 (lung cancer) and H23 (lung cancer), were purchased from ATCC and cultured in RPMI 1640 media supplemented with 10 % fetal bovine serum. Where applicable, cells were treated for 24 h with 100 ng/mL IFN-γ (R&D Systems, Minneapolis, MN). Cell line cells were then detached and spiked into healthy donor (HD) blood, which was then processed per Epic Sciences standard operating procedure [28, 29]. Briefly, red blood cells were lysed using ammonium chloride solution and the remaining nucleated cells were plated onto glass slides at a density of 3 million cells per slide. Slides were then stored in a − 80 °C biorepository until used for immunofluorescence (IF) staining and analysis.

### Patient blood sample processing

Blood samples were collected from 25 bladder cancer patients who consented to an IRB-approved protocol at UCSF. Ten mL of whole blood was collected from each patient in Cell Free DNA BCT tubes (Streck, Omaha, NE) and shipped to Epic Sciences at ambient temperature for processing. Red blood cells were lysed using ammonium chloride solution, and nucleated cells were purified for direct deposition onto glass slides (at a density of 3 million cells per slide) and subsequent storage in the − 80 °C biorepository.

### PD-L1 IF staining and analysis

Slides created from cell line control (CLC)-spiked HD samples or bladder cancer patient samples were subjected to automated IF staining for cytokeratin (CK), CD45 (hematopoietic marker) and PD-L1 (clone E1L3N, Cell Signaling Technology). Stained slides were analyzed with fluorescent scanners and morphology algorithms for the identification of traditional CK^+^ CTCs, CTC clusters, apoptotic CTCs and CK^−^CTCs morphologically distinct from hematological cells [26]. Trained classifiers conducted final classification of CTC subpopulations based on morphological parameters and biomarker expression. CLC slides were stained in parallel with patient samples. Threshold for PD-L1 cell positivity in patient samples was set to 95 % specificity of negative control CLC signal (i.e., 95 % negative control cell line cells lie below threshold).

### Patient sample testing

CTC detection and classification on the Epic Sciences platform has been described previously [28, 29]. In brief, slides created from bladder cancer patient samples underwent automated IF staining for CK, CD45, and PD-L1, and counterstained with DAPI to visualize nuclei. Up to two slides were stained and evaluated per patient sample with fluorescent scanners and morphology algorithms for the identification of CTCs, CTC clusters, and apoptotic CTCs. A more thorough description of CTC types identified on the Epic Sciences platform has been published previously [26]. Briefly, all CTCs are strictly CD45^−^and have intact nuclei, with the exception of apoptotic CTCs, which are defined by their fragmented nuclei. CTCs are classified by the presence or absence of CK staining, and whether they are single CTCs or a CTC cluster. CK^−^CTCs must also exhibit distinctive nuclear morphology compared to neighboring white blood cells (WBCs) consistent with malignant origin. Apoptotic CTCs were defined as CK^+^/CD45^−^CTCs with DAPI pattern of chromosomal condensation and/or nuclear fragmentation and blebbing. A more detailed description of characterization of apoptotic CTCs has been published previously [26, 30]. California-licensed Clinical Laboratory Cytologists conduct final quality control of CTC subpopulation classification.

### Fluorescence in situ hybridization

Slide coordinates of every CTC are recorded during the Epic Sciences CTC enumeration process, from which CTCs were relocated and analyzed by fluorescence in situ hybridization (FISH) for specific genetic alterations. One patient with CK^−^/PD-L1^+^ CTCs was selected and tested for genetic alterations by FISH. Coverslips were removed, IF staining attenuated, and cells were fixed and dehydrated. After dehydration, a probe solution (Cymogen Dx, Irvine, CA) selected for bladder cancer to assess polyploidy and gross genomic alterations in identified CTCs (targeting the CEP3, CEP7, CEP10, 5p15 DNA sequences of interest) was applied to each slide. After hybridization, slides were then washed to remove the unbound probe, counterstained with DAPI, and mounted with an anti-fade mounting medium. Adjacent patient WBCs were used as internal negative controls for endogenous genetic status for each cell analyzed.

### Cell Isolation, amplification, and next-generation sequencing

Non-apoptotic individual CTCs were relocated on the slide based on a mathematical algorithm that converts the original CTC positions (x and y coordinates) computed during the scanning procedure into a new set of x, y references compatible with the Nikon TE2000 inverted immunofluorescent microscope used for cell capture. Single cells were captured using an Eppendorf TransferMan NK4 micromanipulator. Cells were deposited into PCR tubes using 1 μL of TE buffer and single cell whole genome amplification (WGA) was performed using SeqPlex Enhanced (Sigma) according to the manufacturer’s instructions with minor modifications. For patient copy number variation (CNV) analysis, 9 CK^−^/PD-L1^+^ CTCs from one patient were sequenced. Post-WGA, DNA concentrations were determined by UV/Vis. Next-Generation Sequencing (NGS) libraries were constructed using NEBNext Ultra DNA Library Prep Kit for Illumina (NEB) from 100 ng of WGA DNA as per manufacturer recommendation with minor modifications. After NGS library preparation, library concentrations and size distributions were determined with NEBNext Library Quant Kit for Illumina (NEB) and Fragment Analyzer (Advanced Analytical). Equinanomolar concentrations from each library were pooled and sequenced on an Illumina NextSeq 500 using a High Output Paired-End 2 × 150 format (PE 2 × 150).

Genome wide copy number variation analysis was performed using Epic single cell CNV analysis pipeline. Briefly, raw sequencing data (FASTQ) were aligned to hg38 human reference genome from UCSC (http://hgdownload.soe.ucsc.edu/goldenPath/hg38/bigZips/) using Burrows-Wheeler Aligner (BWA, http://bio-bwa.sourceforge.net). Binary alignment map files (BAM) were filtered for quality (MAPQ 30) to keep only the reads that have one or just a few “good” hits to the reference sequence. Hg38 human genome was divided into ~3000 bins of 1 million base pair and reads were counted within each bin for each sample. For each sample, read counts per bin were normalized against white blood cell controls, and the ratios were log2 transformed before plotted against genome positions.

### Clinical data collection and survival analysis

All patients consented to participate in this IRB-approved research study prior to providing peripheral blood samples for analysis. All patient identifiers were removed prior to analysis by Epic Sciences. Clinical data extracted from their charts were maintained and tracked on a secure database. Overall survival was defined as the length of time from the date of the blood draw till death or last follow-up, and was calculated for patients who maintained follow-up at UCSF. Survival analysis was performed using the log-rank test and time-to-event curves were plotted using the Kaplan-Meier method.

## Results

### Assessment of PD-L1 antibody specificity

**Fig. 1:**
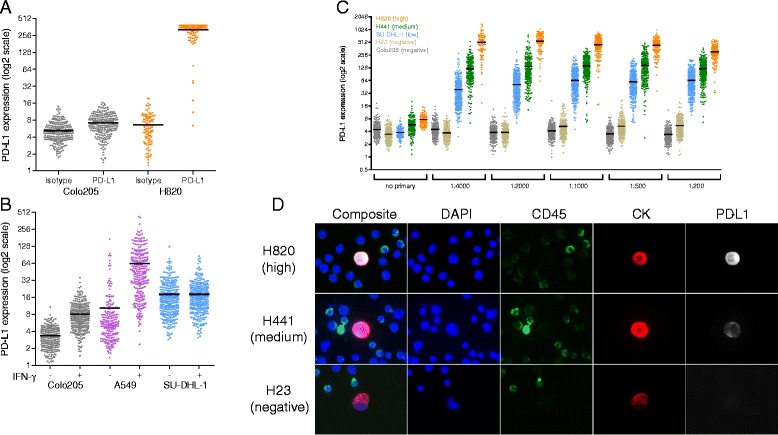
PD-L1 CTC Assay Development (**a**) PD-L1-specific antibody and species-matched isotype control were tested in negative (Colo205) and high (H820) PD-L1-expressing cell lines. Individual cellular PD-L1 IF signal is quantified and plotted. No staining above background was seen with isotype control or in Colo205 stained with anti-PD-L1. **b** IFN-γ treatment increases PD-L1 expression in Colo205 and A549, while SU-DHL-1 is insensitive. **c** PD-L1 antibody was titrated in PD-L1 IF staining of high (H820), medium (H441), low (SU-DHL-1) and negative (Colo205, H23) PD-L1-expressing cell lines to determine assay sensitivity and dynamic range. At the optimal antibody concentration (1:2000 dilution), mean H820, H441 and SU-DHL-1 PD-L1 expression was determined to be 140-, 36- and 13-fold higher than mean background staining in negative controls. **d** Representative images of high, medium and negative PD-L1 expressing cell lines show membrane-localization of PD-L1 IF signal

To evaluate the specificity of the PD-L1 CTC assay, anti-PD-L1 antibody and species-matched isotype controls were tested on high PD-L1 expressing and negative control cell lines (H820 and Colo205, respectively; Fig. 1a). Membrane-localized staining was observed in H820 cells stained with anti-PD-L1, whereas no staining was observed in negative control cell lines or with isotype control antibody. To further evaluate the specificity of the assay for PD-L1 protein, Colo205, A549, and SU-DHL-1 cell lines were treated with interferon gamma (IFN-γ), a known cytokine that induces PD-L1 expression [31] (Fig. 1b). Negative (Colo205) or low (A549, SU-DHL-1) PD-L1-expressing cell lines were selected specifically to observe if IFN-γ was sufficient to up-regulate PD-L1 protein expression. As detected by the PD-L1 CTC assay, IFN-γ treatment increased PD-L1 expression in both Colo205 and A549 cells compared to untreated cells, however, PD-L1 expression in SU-DHL-1 cells remained unchanged. This observed insensitivity to IFN-γ by SU-DHL-1 could be due to suppression of cytokine signaling 3 (SOCS3) protein, known to be highly expressed in SU-DHL-1, which inhibits the cytokine signaling required for IFN-γ mediated PD-L1 induction [32].

### PD-L1 assay development

**Fig. 2:**
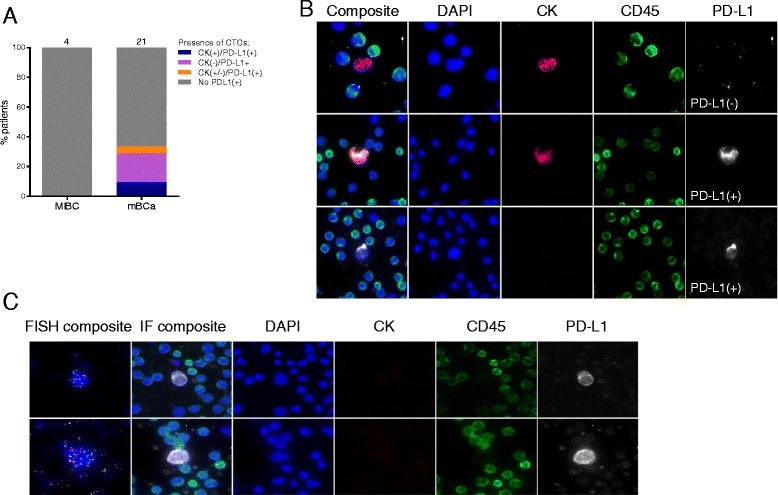
PD-L1 positive CTCs observable in patients with bladder cancer (**a**) Of the 7 total patients with PD-L1^+^ CTCs, two had exclusively CK^+^/PD-L1^+^ CTCs, four patients had CTCs that were exclusively CK^−^/PD-L1^+^, and one had both CK^−^and CK^+^/PD-L1^+^ CTCs. Further breakdown of CK^+^/PD-L1^+^ and CK^−^/PD-L1^+^ CTCs detected by tumor subtype and staging indicates that inclusion of CK^−^ CTCs substantially increased sensitivity of PD-L1^+^ CTC detection. **b** Representative images of CK^+^/PD-L1^+^ and CK^−^/PD-L1^+^ patient CTCs are shown. **c** Representative FISH images of CK^−^/PD-L1^+^ cells demonstrate gross genomic instability and polyploidy using DNA probes for CEP3 (*aqua*), CEP7 (*orange*), CEP10 (*green*) and 5p15 (*red*). 29/33 (88 %) CK^−^/PD-L1^+^ cells assessed from one individual patient with high CTC burden were observed to have at least one abnormality determined by FISH

Anti-PD-L1 titration curves were generated with cell line controls expressing high (H820), medium (H441), low (SU-DHL-1) and negative (H23, Colo205) levels of PD-L1 (Fig. 2a). At the optimal antibody condition (1:2000 dilution), relative mean IF PD-L1 signal in H820, H441, SU-DHL-1 was detected at 140-, 36-and 13-fold, respectively, over baseline levels in negative control Colo205 (Fig. 1c). As expected, PD-L1 staining in positive cell lines was observed to be enriched in the plasma membrane (Fig. 1d) [33].

### Patient characteristics

#### Demographics

Blood was obtained from 25 patients with bladder cancer from May 2014 to January 2016. Follow-up data for clinical outcomes was available for 19 patients. This cohort of patients represented a broad range of burden of disease. Overall, 17 men and 8 women participated in the study. The median age of all participants was 67 (range: 43 – 89), 4 patients had MIBC at the time of draw and 21 had mBCa. Fifteen of these patients had received prior chemotherapy, with at least 12 receiving one or more cisplatin-based regimens. Median time to blood draw from diagnosis was 1075 days. Please refer to Table 2 for median time to blood draw from diagnosis and prior therapies received. Of note, two patients who contributed samples had prior malignancies. One patient (B-026) had a history of BRCA2 positive breast cancer with no evidence of disease since 1993. The second patient (B-011) developed acute myeloid leukemia (AML) months after his CTCs were drawn. This patient is included in the analysis as the CTCs were CD45^−^, while cells from AML were found to be uniformly CD45^+^; FISH analysis performed on these CD45^−^ CTCs confirmed genomic alterations consistent with bladder cancer, further discussed below. See Table 1 for summary of patient demographics.

**Table 1:** Baseline patient demographics

Number of patients	25
Age, y	
Median	67
Min-max	43–89
Sex, *n* (%)	
Male	17 (68.0)
Female	8 (32.0)
Extent of disease, *n* (%)	
MIBC	4 (16.0)
mBCa	21 (84.0)
Prior chemotherapy, *n* (%)	15 (60.0)
Follow-up status, *n* (%)	
Deceased	11 (44.0)
Alive	8 (32.0)
No follow-up	6 (24.0)
Median days to blood draw	1075
Survival after CTC draw, days	
Median	167
Min-max	40–599

Abbreviations: *Y*, years, *Min*, minimum, *Max*, maximum, *n*, number of patients per category

#### CTC Detection

CK^+^ single CTCs were detected in 13/25 (52 %) patient samples. CK^+^ CTC Clusters were detected in 6/25 (24 %), apoptotic CTCs were detected in 13/25 (52 %), and CK^−^ CTCs were detected in 14/25 (56 %) patient samples. Combined, 20/25 (80 %) patient samples had detectable CTCs of all subtypes. Interestingly, 3 of 4 patients with MIBC had detectable CTCs (one patient had CK^−^CTCs, one with CK^+^ CTCs, and one with apoptotic CTCs).

#### PD-L1 Expression on CTCs

Of the 20 patients with detectable CTCs, 7/20 (35 %) had CTCs with PD-L1 positivity. Of these, five patients had CK^−^/PDL1^+^ CTCs, with four of these patients having PD-L1 positivity exclusively on CK^−^ CTCs. Patients with PD-L1 expression detectable on CTCs all had metastatic bladder cancer. See patient sample CTC summary in Table 2 and Fig. 2a. Thus far, seven patients have received PD-1 targeting therapy-one received it prior to CTC collection, the remaining started therapy after blood collection.

**Table 2:** CTCs detected in patient samples

						CTC subtype/mL			
Patient ID	Extent of disease at time of draw	Days from diagnosis to draw	Cycles of chemo prior to draw	CK^+^	CK^+^ Clusters	CK^-^	CK^-^ Clusters	Apoptotic	PD-L1+ (%)^a^
B-002	MIBC	4882	2	0.0	0.0	1.1	0.0	0.0	0.0 (0.0)
B-003	MIBC	59	0	0.0	0.0	0.0	0.0	1.8	0.0 (0.0)
B-015	MIBC	58	0	1.0	0.0	0.0	0.0	1.5	0.0 (0.0)
B-018	MIBC	150	4^b^	0.0	0.0	0.0	0.0	0.0	0.0 (0.0)
B-001	mBCa	55	0	3.3	0.0	2.5	0.0	0.0	0.0 (0.0)
B-004	mBCa	553	4^b^	0.0	0.0	5.0	0.0	0.0	0.0 (0.0)
B-005	mBCa	225	4^b^	34.7	0.3	0.7	0.0	12.5	0.3 (0.9)
B-006	mBCa	27	0	7.6	1.5	1.5	0.0	0.0	0.0 (0.0)
B-007	mBCa	162	0	0.0	0.0	0.8	0.0	1.7	0.0 (0.0)
B-010	mBCa	1513	9	1.5	0.0	3.7	0.0	0.0	0.7 (14.3)
B-011	mBCa	633	0	153.2	6.4	2002.1	38.3	0.0	972.3 (44.2)
B-012	mBCa	4606	1	5.5	0.0	5.2	0.0	6.7	0.6 (5.7)
B-013	mBCa	423	5^b^	0.0	0.0	0.0	0.0	0.0	0.0 (0.0)
B-014	mBCa	43	2^b^	0.0	0.0	3.3	0.0	1.6	0.0 (0.0)
B-016	mBCa	939	4^b^	11.9	0.6	5.8	1.0	1.0	0.0 (0.0)
B-017	mBCa	109	0	0.0	0.0	0.0	0.0	0.8	0.0 (0.0)
B-019	mBCa	163	4^b^	0.0	0.6	1.3	0.0	0.0	1.3 (66.7)
B-020	mBCa	815	8^b^	4.2	0.0	0.0	0.8	1.7	0.0 (0.0)
B-022	mBCa	7119	20^b^	4.3	0.0	22.9	0.0	5.7	12.9 (47.4)
B-023	mBCa	444	5	0.0	0.0	0.0	0.0	0.0	0.0 (0.0)
B-025	mBCa	1659	0	76.6	4.3	2.1	0.0	1.1	1.1 (1.3)
B-026	mBCa	724	5^b^	4.2	0.0	0.0	0.0	2.1	0.0 (0.0)
B-027	mBCa	271	4^b^	0	0	0	0	0	0.0 (0.0)
B-028	mBCa	247	0	0.9	0	0	0	1.8	0.0 (0.0)
B-029	mBCa	996	0	0	0	0	0	0	0.0 (0.0)

^a^ Includes CK^+^ and CK^−^CTCs

^b^ indicates patient received at least one cisplatin regimen

### CK^−^ PDL1^+^ CD45^−^CTCs have gross genomic aberrations

To further assess the specificity of the PD-L1 assay in patient samples, we analyzed CK^−^/CD45^−^/PD-L1^+^ CTCs from two metastatic bladder cancer patients for genomic abnormalities. Patient selection was determined by the ability to perform FISH analysis, which excluded those requiring PDL1 amplification during processing of the samples. Thirty-three CK^−^/CD45^−^/PD-L1^+^ CTCs detected in one mBCa patient (B-011) were analyzed by FISH for genetic alterations commonly associated with bladder cancer. As shown in Fig. 2c, the presence of these genetic abnormalities in identified CTCs is consistent with malignant origin. See detailed results in Table 3.

**Table 3:** Assessment of CK-/PD-L1+ CTCs for genetic alterations by FISH

FISH status	Pt 11 CK^-^CTCs (*N* = 33)
No abnormalities, *n* (%)	4 (12.1)
At least 1 abnormality, *n* (%)	29 (87.9)
All abnormalities, *n* (%)	17 (51.5)

Abbreviations: *N*, number of CTC analyzed for genetic alterations by FISH, *n*, number of CTCs per category

**Fig. 3:**
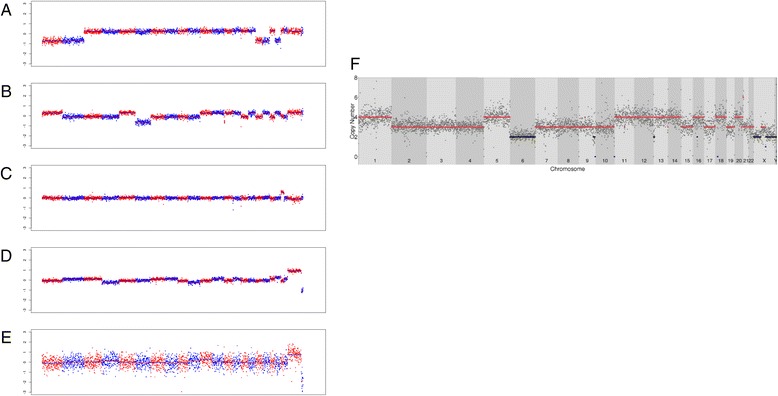
Genomic heterogeneity of bladder cancer CTCs. Plots of whole genome CNV profiles of five CK^−^/CD45^−^/PD-L1^+^ CTCs from patient B-022 (**a**-**e**). X axis: chromosomes displayed as from chromosome 1 to 22, X and Y (from left to right, shifted by *red* and *blue* color); Y axis: normalized log2 transformed ratio of copy number of test sample over that of WBC control. Five CTCs show various genomic aberrations **a** loss of chromosome 1, 2, 17, 18, and 20; **b** loss of chromosome 6; **c** gain of chromosome 21; **d** and **e** gain of chromosome X and loss of chromosome Y. **f** Ploidy analysis for genomic aberrations from NSG seen in CTC (**b**), predicted ploidy = 3.25

Nine CK^−^/CD45^−^/PD-L1^+^ CTCs detected in a different patient with metastatic disease (B-022) were further assessed for CNV using NGS. Read count analysis of these CTCs are provided in Additional file 1: Figure S1. Five of nine (56 %) CTCs demonstrated a significant number of genomic aberrations in chromosomal copy number changes, including chromosomes 1, 2, 6, 17, 18, 20, 21, X and Y (Fig. 3a–e). Figure 3f depicts the ploidy analysis of genomic aberrations seen in the CTC evaluated in Fig. 3b. As seen, this shows numerous amplifications and deletions within multiple arms and chromosomes. Interestingly, chromosome 6 and Y appear to be diploid, while the remainder of the chromosomes demonstrate some level of ploidy abnormality. Evaluating these results against copy number data available from The Cancer Genome Atlas (TCGA) cohort of 412 MIBC patients, we found some concordant losses in chromosomes 1, 2, 6, 17, and 18. These genomic aberrations provide supporting data that the cells identified were of malignant origin.

### High circulating PD-L1^+^ CD45^−^CTC burden and overall survival

Follow-up survival data was available for 19 of 25 patients. High burden of PD-L1^+^ CTC is defined as >1 PD-L1^+^ CTC/mL (*n* = 4). Low burden of PD-L1^+^ CTC is defined as greater than 0, but less than 1 PD-L1^+^ CTC/mL (*n* = 3). PD-L1^+^ negative staining was defined as 0 PD-L1^+^ CTCs/mL (*n* = 12). Recent data presented by Boffa, et al. at the American Society of Clinical Oncology (ASCO 2016) meeting, which utilized the Epic PD-L1 assay to demonstrate higher burden of PD-L1^+^ CTCs is a poor prognostic factor in lung cancer compared those patients with the highest burden of PD-L1^+^ CTCs to low/negative PD-L1^+^ CTCs. Our analysis used a similar approach evaluating highest burden of PD-L1^+^ CTCs (>1/mL, *n* = 4) to the rest of the cohort (*n* = 15). While no statistically significant conclusions could be drawn from this data, patients with higher circulating PD-L1^+^ CTC burden had a shorter median overall survival (194 vs. 303 days, log-rank *p* = 0.416) compared to those with low circulating PD-L1+ CTC burden (Additional file 2: Figure S2A).

Of the seven patients who received PD-1 checkpoint immunotherapy, follow-up survival data was available for 5 patients. One patient passed away after one cycle of therapy. The remaining four patients received at least three cycles of therapy. Two of four patients had detectable CTCs (B-025 and B-028). Only patient B-025 exhibited PD-L1+ (1.3 %) CTCs. The patient demonstrated progression on PD-1 immunotherapy on radiographic assessment after cycle 5 of therapy and was discontinued. The other three patients who lacked PD-L1+ CTCs also demonstrated radiographic progression on PD-1 immunotherapy after 3, 6, and 8 cycles of therapy, respectively.

### Apoptotic CTCs and overall survival

Thirteen out of 25 patients (52 %) were detected to have apoptotic CTCs. Two of four patients with MIBC had detectable apoptotic CTCs (B-003 = 1.8, B-015 = 1.5) and 11/16 (69 %) mBCa patients with detectable CTCs had apoptotic CTCs. Kaplan-Meier analysis of all 13 patients with apoptotic CTCs suggests a trend towards improved overall survival in patients with apoptotic CTCs (Hazard ratio = 0.4, 0.12–1.31; *p* = 0.159). See Additional file 2: Figure S2B.

## Discussion

In this study, we demonstrate the ability to detect PD-L1 positivity both in cell lines spiked into human blood as well as in bladder cancer CTCs processed on the Epic Sciences platform. Pre-clinically, using cell lines with known PD-L1 expression, we observed assay specificity for PD-L1 expression by IF staining. Furthermore, expression was found to be 140-fold higher in H820 (high expressing) cells as compared to negative controls, indicative of high dynamic range and assay sensitivity. This is further supported by detection of upregulated PD-L1 expression on Colo205 and A549 cell lines treated with IFN-γ.

We evaluated the clinical utility and feasibility of the Epic Sciences PD-L1 CTC assay using 25 bladder cancer patient samples of various stages (MIBC and mBCa). While CTCs were detected in 3 of the 4 patients with MIBC, PD-L1 expression was not identified in this small sub-cohort of patients. PD-L1^+^ CTCs were detected in 7/20 (35 %) patients with mBCa, and four of these patients expressed PD-L1 exclusively on CK^−^ CTCs. To confirm that these cells were truly CTCs, CK^−^/PD-L1^+^ CTCs from one patient were evaluated using a bladder cancer FISH probe panel and CK^−^/PD-L1^+^ CTCs from another patient were assessed for CNV by NGS. Both of these methods found genomic aberrations in CTCs consistent with malignant origin. CNV analysis on 5 of 9 CTCs that underwent NGS showed marked chromosomal copy number variations with ploidy analysis of one cell revealing a high level of aberrancy. Four of the nine cells did not exhibit a large number of chromosomal copy number variations (see Additional file 2: Figure S2). The heterogeneity of aberrancies found in these cells is consistent with prior findings of intratumoral DNA ploidy heterogeneity described in various tumor types, including bladder cancer [34–36] and highly supports malignant origin. The finding of patients with exclusively CK^−^/PD-L1^+^ cells is intriguing as it could suggest that cells undergoing mesenchymal differentiation and metastasis may escape immune surveillance potentially via expression of PD-L1. However, this finding requires confirmation in a larger cohort. This also points to the utility of using a CTC enrichment technique that does not require CK expression.

It has previously been demonstrated in bladder cancer and other solid tumors using tissue biopsy staining that patients fare worse if their tumors are able to evade the immune system [37]. While these patient samples represent a small, cross-sectional cohort rather than a prospective controlled trial, it is worth noting that those with the highest PD-L1^+^ CTC burden (>1/mL) had a shorter overall survival from the time of the CTC draw. Furthermore, evaluation of apoptotic CTC counts demonstrated a trend toward shorter survival for those with fewer apoptotic CTCs. Fewer apoptotic cells have been observed in patients with metastatic breast cancer compared to those with early stage disease, suggesting a correlation between lack of apoptosis with cell survival and an aggressive phenotype [38]. Of note, the time of CTC draw was highly variable across our patient population and represented snapshots of a wide range of burden of disease and prior therapies. Some patients were actively undergoing chemotherapy at the time of their draw, which could influence the burden of total and apoptotic CTCs detected. The aim of this study was to demonstrate feasibility and consistency in detecting PD-L1^+^CTCs in bladder cancer and was not powered to evaluate survival benefits. Further evaluation and optimization in a larger and more uniform cohort with appropriate power and design is warranted to better evaluate the association of PD-L1 expression and apoptotic CTCs with survival.

Biomarkers to predict response to PD-1-directed therapies are far from established. Higher PD-L1 expression in solid tumor biopsy samples is associated with response to pembrolizumab in non-small cell lung cancer [20] and correlates with response to therapy in other indications [14, 39, 40]. However, a significant portion of PD-L1^−^ patients also respond to therapy. Recent multifocal genetic and proteomic analyses of regions within tumors have revealed levels of spatial heterogeneity in several cancer types that might limit the interpretation of solid tumor biopsies [41–45]. Due to tumor heterogeneity, smaller sample size or intratumoral location of the biopsy site may yield a false negative tissue assessment of PD-L1. Similarly, PD-L1 expression is thought to be dynamic and can vary in response to interferon levels and possibly other factors, making the reliability of a static assessment on a limited tumor tissue sample questionable. While it would be challenging to monitor expression via serial tumor tissue biopsies, it is much more feasible to monitor expression of this dynamic marker via serial CTC assessment from whole blood. It may even be possible to detect PD-L1^+^ CTCs in patients who are PD-L1^−^on tissue biopsy. The correlation of CTC PD-L1 status with paired tumor tissue samples and the implications of discordant expression was not pursued in this study, but will be important for future assessment. Similarly, five evaluable patients received PD-1 targeting therapy after blood was drawn for analysis of CTCs. One patient had a rapid decline after therapy and the remaining 4 patients demonstrated radiographic progression after 3, 6, and 8 cycles of PD-1 checkpoint immune therapy. Serial blood draws were not performed in this study. Therefore there was an insufficient sample size to assess the prognostic value of PD-L1^+^ CTCs in patients receiving checkpoint inhibitors. This would be best explored in a prospective study with a larger cohort of patients with similar disease burden receiving PD-1 or PD-L1 checkpoint therapy.

Another limiting factor in the analysis of PD-L1 expression in solid tumor samples is the lack of standardization of PD-L1 immunohistochemical assays and their respective positivity thresholds [13, 14]. CTCs provide a minimally-invasive sampling method that could prove useful for prognostication of therapeutic benefit through longitudinal monitoring and measurement of pharmacodynamic changes in CTC counts and/or changes of CTC PD-L1 expression. The Epic Sciences CTC platform utilizes a central laboratory for consistent quality with a central biorepository for retrospective analyses of biomarkers on morphologically intact CTCs. Analytical validation studies of Epic’s CTC platform have been published [26]. In addition, this platform has previously been compared to the FDA approved CellSearch platform (Janssen Diagnostics, NJ, USA) demonstrating consistent, if not increased sensitivity in the detection of CTCs [27]. Using this technology, repeat sampling of patients utilizing CTCs is both feasible and amenable to pharmacodynamic biomarker development to identify not only patients that might respond, but those who *are* responding to therapy.

## Conclusions

Our findings demonstrate the ability to detect and quantify PD-L1 protein on bladder cancer patients’ CTCs using an assay with specificity and sensitivity demonstrated in CTC surrogate cell lines. Exploratory analysis of survival data suggests a trend towards improved survival in those with low PD-L1 expression or with higher burden of apoptotic CTCs. While the data presented here are compelling, it should be emphasized that this study is descriptive, represents a small sample size, and requires validation with a larger, prospective study encompassing a broader patient population that is appropriately powered to evaluate survival benefits. Nonetheless, these data provide initial support for broader development of CTC PD-L1 expression. With further study, PD-L1 expression on CTCs isolated from peripheral circulation has the potential to become a new prognostic and predictive biomarker with which to stratify treatments for patients and possibly predict response to immunotherapy in bladder cancer.

## Additional files

Additional file 1: Sequencing read counts for 10 CTCs from two patients with metastatic bladder cancer undergoing NGS. (PPTX 48 kb) Additional file 2: (A) Kaplan-Meier curve of OS for patients with high (solid line) and low (dotted line) PD-L1^+^ CTC burden (high burden ≥ 1 PD-L1^+^ CTC/mL). (B) Kaplan-Meier curve of OS for patients with apoptotic CTCs (dotted line) and without apoptotic CTCs (solid line). (PPTX 71 kb)

## Abbreviations

BAM: Binary Alignment Map
CK: Cytokeratin
CLC: Cell line control
CNV: Copy number variation
CTC: Circulating tumor cell
FISH: Fluorescence in situ hybridization
HD: Healthy donor
IF: Immunofluorescence
IFN-γ: Interferon gamma
mBCa: Metastatic bladder cancer
MIBC: Muscle-invasive bladder cancer
NGS: Next generation sequencing
PD-1: Programmed death 1
PD-L1: Programmed death ligand 1
TCGA: The cancer genome atlas
WBC: White blood cell
WGA: Whole genome amplification
