# Supplementary material for: Programmed death-ligand 1 (PD-L1) characterization of circulating tumor cells (CTCs) in muscle invasive and metastatic bladder cancer patients
Source: BMC Cancer. 2016 Sep 22;16:744. doi: 10.1186/s12885-016-2758-3 (PMC5034508; doi:10.1186/s12885-016-2758-3)
Supplement: Additional file 1: — Sequencing read counts for 10 CTCs from two patients with metastatic bladder cancer undergoing NGS. (PPTX 48 kb) [file 12885_2016_2758_MOESM1_ESM.pptx]

## Slide 1
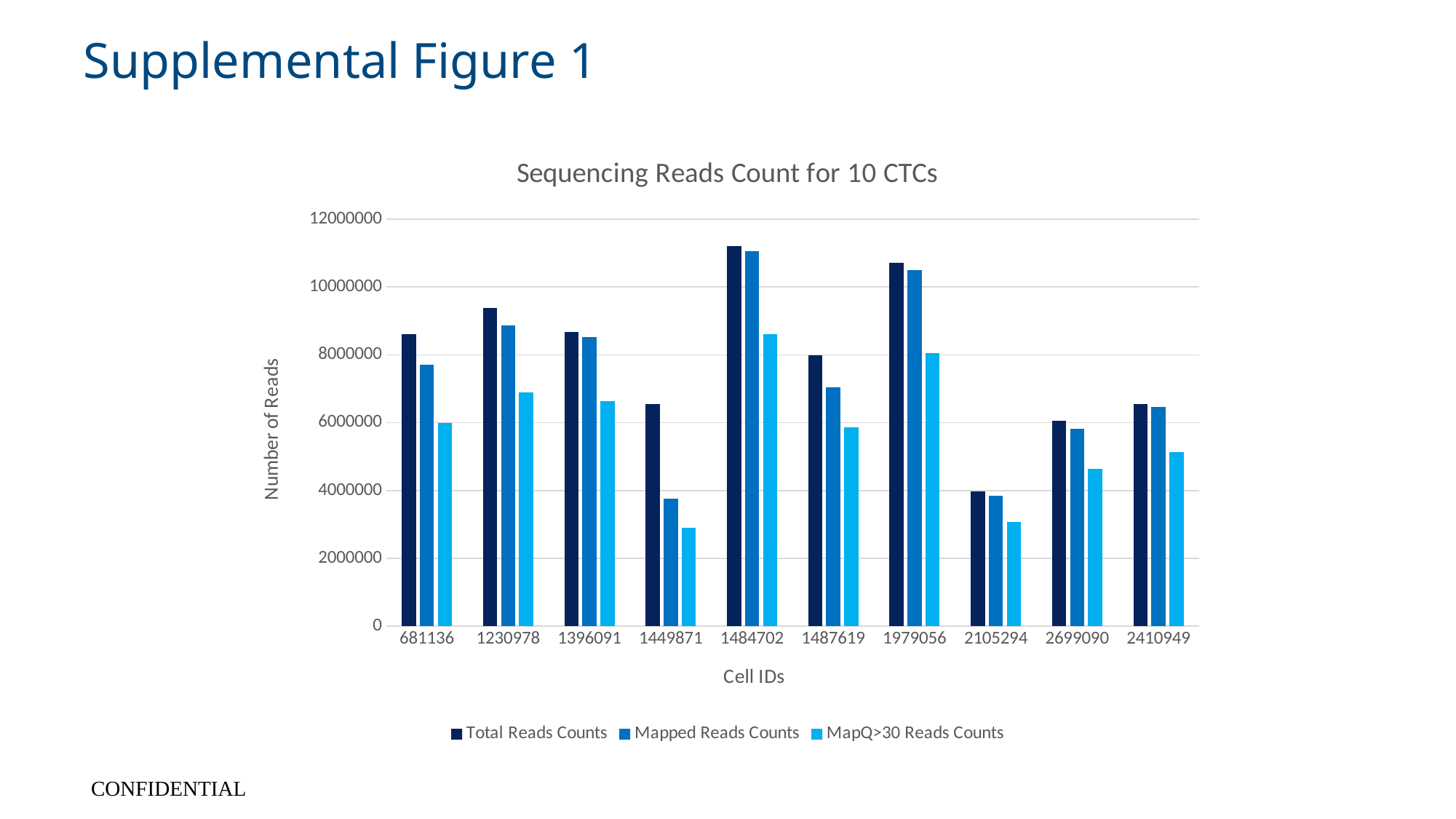

# Supplemental Figure 1
### Chart: Sequencing Reads Count for 10 CTCs
| Category | Total Reads Counts | Mapped Reads Counts | MapQ>30 Reads Counts |
|---|---|---|---|
| 681136.0 | 8605066.0 | 7712023.0 | 5981648.0 |
| 1.230978E6 | 9373324.0 | 8864568.0 | 6889596.0 |
| 1.396091E6 | 8670081.0 | 8533866.0 | 6637113.0 |
| 1.449871E6 | 6549978.0 | 3748530.0 | 2902058.0 |
| 1.484702E6 | 11201864.0 | 11056405.0 | 8621285.0 |
| 1.487619E6 | 7984180.0 | 7050926.0 | 5867283.0 |
| 1.979056E6 | 10724833.0 | 10491696.0 | 8051752.0 |
| 2.105294E6 | 3983863.0 | 3850083.0 | 3062687.0 |
| 2.69909E6 | 6049939.0 | 5820867.0 | 4638955.0 |
| 2.410949E6 | 6553744.0 | 6463134.0 | 5126475.0 |CONFIDENTIAL
1
