# Supplementary figures and images for: Programmed death-ligand 1 (PD-L1) characterization of circulating tumor cells (CTCs) in muscle invasive and metastatic bladder cancer patients
Source: BMC Cancer. 2016 Sep 22;16:744. doi: 10.1186/s12885-016-2758-3 (PMC5034508; doi:10.1186/s12885-016-2758-3)

## Slide 1
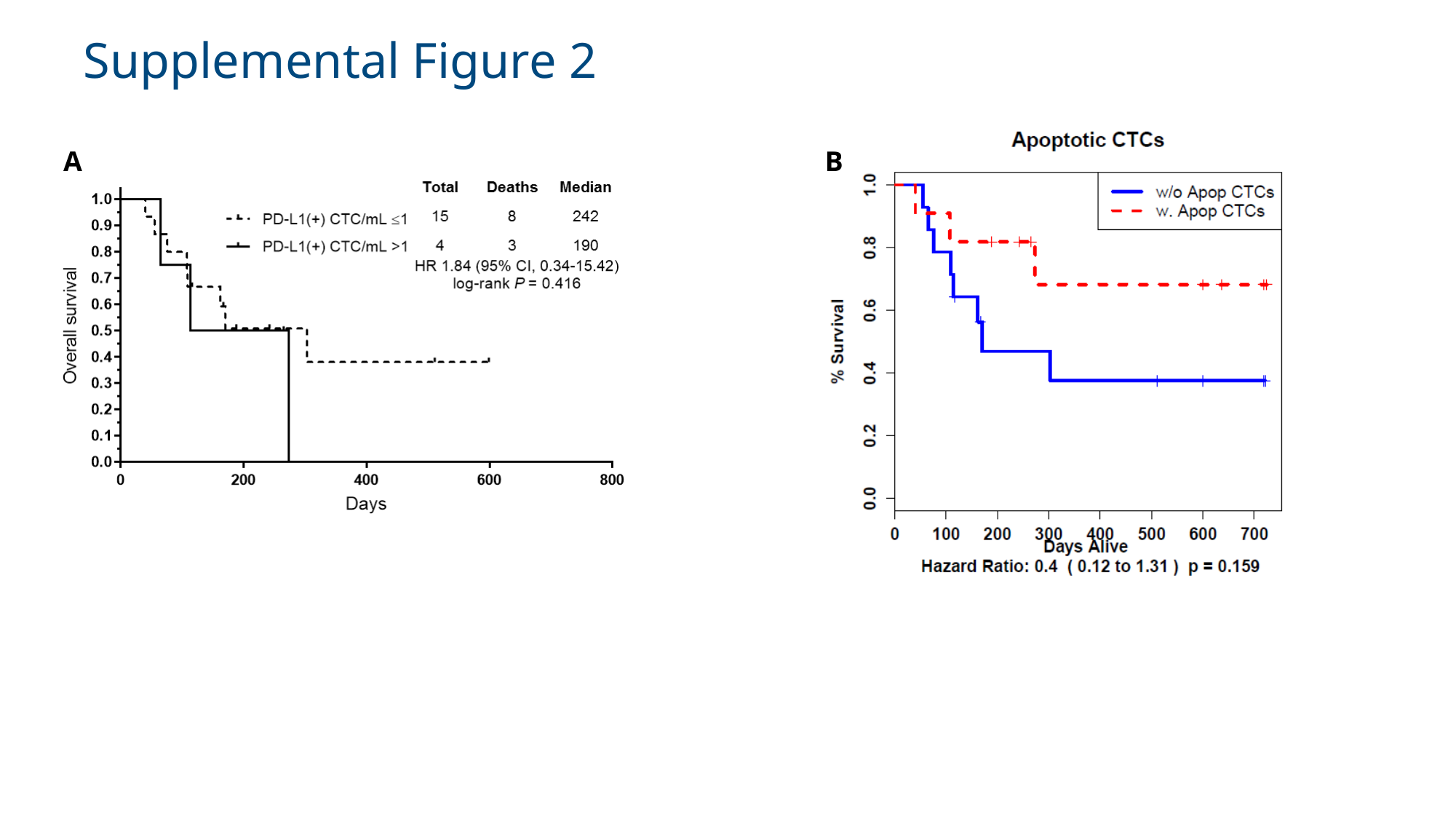

# Supplemental Figure 2
A
B

Supplement: Additional file 2: — (A) Kaplan-Meier curve of OS for patients with high (solid line) and low (dotted line) PD-L1+ CTC burden (high burden ≥ 1 PD-L1+ CTC/mL). (B) Kaplan-Meier curve of OS for patients with apoptotic CTCs (dotted line) and without apoptotic CTCs (solid line). (PPTX 71 kb) [file 12885_2016_2758_MOESM2_ESM.pptx]
